# Exploring the Genetic Landscape of Childhood Glaucoma

**DOI:** 10.3390/children11040454

**Published:** 2024-04-09

**Authors:** Yang Pan, Takeshi Iwata

**Affiliations:** National Institute of Sensory Organs, NHO Tokyo Medical Center, Tokyo 152-8902, Japan; yang.pan@kankakuki.jp

**Keywords:** childhood glaucoma, genetic, *CYP1B1*, *LTBP2*, *TIE2*, *ANGPTI*, *FOXC1*, *MYOC*

## Abstract

Childhood glaucoma, a significant cause of global blindness, represents a heterogeneous group of disorders categorized into primary or secondary forms. Primary childhood glaucoma stands as the most prevalent subtype, comprising primary congenital glaucoma (PCG) and juvenile open-angle glaucoma (JOAG). Presently, multiple genes are implicated in inherited forms of primary childhood glaucoma. This comprehensive review delves into genetic investigations into primary childhood glaucoma, with a focus on identifying causative genes, understanding their inheritance patterns, exploring essential biological pathways in disease pathogenesis, and utilizing animal models to study these mechanisms. Specifically, attention is directed towards genes such as *CYP1B1* (cytochrome P450 family 1 subfamily B member 1), *LTBP2* (latent transforming growth factor beta binding protein 2), *TEK* (TEK receptor tyrosine kinase), *ANGPT1* (angiopoietin 1), and *FOXC1* (forkhead box C1), all associated with PCG; and *MYOC* (myocilin), associated with JOAG. Through exploring these genetic factors, this review aims to deepen our understanding of the intricate pathogenesis of primary childhood glaucoma, thereby facilitating the development of enhanced diagnostic and therapeutic strategies.

## 1. Introduction

Glaucoma constitutes a group of chronic and progressive optic neuropathies distinguished by progressive visual field loss and optic disc damage, standing as the leading cause of irreversible blindness globally [1]. It can occur at all ages, with early-onset cases (before 40 years) typically displaying Mendelian inheritance patterns, while adult-onset forms (after 40 years) exhibit inheritance as complex traits [2]. Generally, mutations in genes causing early-onset glaucoma are rare but have significant biological effects, whereas variants contributing to adult-onset forms are more common with minor effects [3]. Childhood glaucoma, denoted by the onset occurring before 18 years of age, represents a subset within the early-onset category [4]. 

Various definitions and classification systems have been used to describe different subtypes of childhood glaucoma, lacking consensus until recently. To address this situation, the Childhood Glaucoma Research Network (CGRN) has developed a classification system, adopted by the World Glaucoma Association and the American Board of Ophthalmology [5]. Diagnosis of childhood glaucoma requires at least two of the following criteria: (1) intraocular pressure (IOP) of 21 mmHg or higher; (2) glaucomatous optic nerve damage, such as increased cupping, focal notching, or cup-to-disc asymmetry of 0.2 or more between both eyes; (3) corneal changes, such as an increased corneal diameter or Haab striae; or (4) visual field defects consistent with glaucomatous optic nerve damage. According to the CGRN system, childhood glaucoma is categorized as primary or secondary, with primary glaucoma being further subdivided into primary congenital glaucoma (PCG) and juvenile open-angle glaucoma (JOAG) [5]. PCG typically presents with characteristic signs in neonates and infants, while JOAG is often asymptomatic and may be detected incidentally or through family screening [6].

The incidence of childhood glaucoma varies among different populations and subtypes. Six published reports have consistently identified PCG as the prevalent common subtype, with studies from Egypt (55.07% from 207 samples [7]; 68.25% from 652 samples [8]), the USA (19.2% from 205 samples [9]), Brazil (43.95% from 496 samples [10]), India (35.27% from 275 samples [11]), and Australia/New Zealand (57.59% from 290 samples [4]). In contrast, two studies from Thailand and Boston have identified PCG (20.8% in Thailand; 29% in Boston) as the second most common subtype [12]. These findings underscore the variability in prevalence among different geographic populations and suggest potential genetic influences.

Primary childhood glaucoma is typically caused by genetic variants following a Mendelian pattern of inheritance [4]. The important genes implicated in this condition include *CYP1B1* (cytochrome P450 family 1 subfamily B member 1), *LTBP2* (latent transforming growth factor beta binding protein 2)*, FOXC1* (forkhead box C1), *TEK* (TEK receptor tyrosine kinase, also known as *TIE2*), and *ANGPT1* (angiopoietin 1) for PCG; and *MYOC* (myocilin) for JOAG. These gene variants typically exhibit strong penetrance but variable expressivity, contributing to a diverse phenotypic spectrum and overlap between clinical entities. This review aims to explore recent advancements and new insights into the genetics underlying Mendelian forms of primary childhood glaucoma. While the precise functions of these genes and the effects of their variants on health and disease remain incompletely understood, these discoveries offer valuable insights for enhancing clinical diagnoses in childhood glaucoma, despite the challenges in accurately establishing such diagnoses.

## 2. Primary Congenital Glaucoma (PCG)

PCG represents a severe manifestation of the disease, characterized by infant or early-childhood (before three years old) ocular hypertension, enlarged eye globes, and optic neuropathy, often leading to vision loss and blindness despite treatment [13]. Notably, PCG constitutes 18% of children enrolled in institutions for blind people globally [14]. Although PCG occurs across all ethnic groups, its incidence varies based on ethnic background, with rates from 1:1250 in endogamous populations to 1:30,000 in ethnically diverse populations [15,16]. Typically inherited as an autosomal recessive trait, PCG is more prevalent in regions and cultures that practice consanguinity. Linkage analysis studies have identified five loci (*GLC3A* (2p22–p21) [17], *GLC3B* (1p36.2–p36.1) [18], *GLC3C* (14q24.3) [19], *GLC3D* (14q24) [20], and *GLC3E* (9p21) [21]), and three genes (*CYP1B1*, *LTBP2*, and *TEK*) located at these loci [14,22,23,24]. Additionally, genomic analyses, including whole-exome and direct Sanger sequencing analyses, have demonstrated that mutations in the *ANGPT1* and *FOXC1* genes also contribute to PCG [25,26]. 

### 2.1. CYP1B1

#### 2.1.1. *CYP1B1* Mutations in PCG

The *CYP1B1* gene is located on chromosome 2p22–21 and consists of only three exons, including a non-coding exon 1 [27]. It was the first gene to be genetically linked to PCG, with its mutations being the most frequently identified genetic defect causing autosomal recessive PCG globally. To date, over 150 distinct mutations of *CYP1B1* have been identified in PCG cases worldwide. The prevalence of *CYP1B1* mutations in PCG varies significantly across different populations. Specifically, *CYP1B1* mutations have been reported in approximately 80–100% of Saudi Arabians and Slovakian Rom populations [28], as well as in 70% of Iran [29], 50% of Brazil [30], and 44% of India [31]. Conversely, the incidence of PCG caused by *CYP1B1* mutations is lower in Indonesian, Japanese, and Han Chinese populations, standing at 33.3%, 20%, and 17.2%, respectively [32]. Consanguineous marriages, known for their increased risk of developing autosomal recessive disorders [33], contribute to the prevalence of *CYP1B1* mutations causing autosomal recessive PCG. This practice is particularly prevalent in Muslim-majority countries in North and sub-Saharan Africa, the Middle East, and parts of West, Central, and South Asia [34,35]. Therefore, the variation in the incidence of *CYP1B1* mutations in PCG can be largely attributed to the prevalence of consanguineous marriage within these populations.

**Figure 1:**
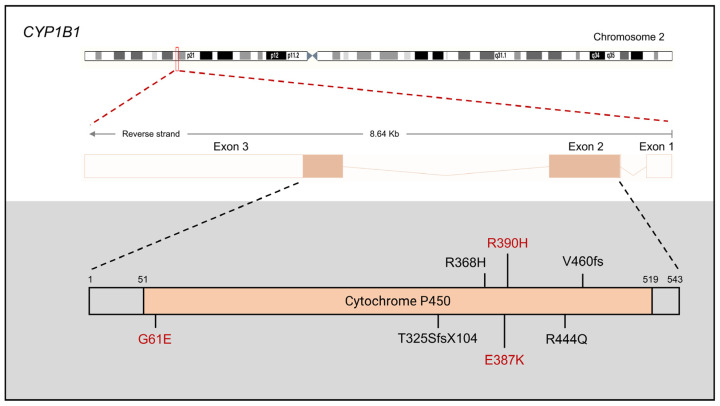
Gene structure of *CYP1B1* and the locations of the most prevalent mutations associated with primary congenital glaucoma (PCG). The most common mutations reported across different populations are indicated in red, while mutations specific to certain populations are shown in black.

Moreover, the most prevalent *CYP1B1* mutation varies among different populations (Figure 1). The point mutation *CYP1B1* p. Gly61Glu (G61E) is the predominant mutation in Saudi Arabia, Portugal, Vietnam, Morocco, and Iran [36]. Conversely, the second point mutation, p. Glu387Lys (E387K), is the most common mutation observed in Slovakian Rom and Hungarian populations [36]. In Han Chinese and Pakistani populations, p.Arg390His (R390H) stands as the most frequent mutation, causing a severe form of PCG that is resistant to surgical interventions and medications [37]. Additionally, certain mutations serve as the most common mutation within specific populations but not in others. For instance, *CYP1B1* p.Arg444Gln (p.R444Q) predominates in Japanese populations [38], c.970_971dupAT (p.T325SfsX104) is prevalent in Koreans [39], g.8214_8215delAG (p.Val460fs) is common in Brazilians [40], and p.R368H is notable among Indians [40]. A mixed European study identified 31 *CYP1B1* mutations in 56 PCG patients (34.7%), with p.E387K being the most prevalent mutation [41]. However, the exact impact of *CYP1B1* disruption in various European populations remains unclear due to limited samples, necessitating further research.

#### 2.1.2. CYP1B1 Protein Function in PCG

The CYP1B1 protein belongs to the cytochrome P450 (CYP) enzyme family, which plays a crucial role in catalyzing oxidation reactions of various organic substrates [14]. It is expressed in numerous adult and fetal extrahepatic tissues, including brain, kidney, prostate, breast, and ocular tissues [42]. Although the precise function of CYP1B1 in the eye remains elusive, several pathways regulated by this protein have been implicated in PCG. 

First, CYP1B1 is integral to properly developing trabecular meshwork (TM), a critical tissue for controlling IOP in relation to PCG pathogenesis. The strong expression of CYP1B1 in ocular tissues underscores its importance in normal eye development and function in both mice and humans [43,44]. Mice lacking Cyp1b1 exhibit anomalies such as small or absent Schlemm’s canal (SC), TM fibers, and angle closure [45]. The precise mechanism by which CYP1B1 contributes to TM development remains under investigation; however, some studies show its involvement in modulating oxidative stress and extracellular matrix proteins [46]. 

Secondly, the role of CYP1B1 in angiogenesis is noteworthy. Its expression in neurovascular structures, including astrocytes, suggests an involvement in postnatal retinal vascular development and pathological neovascularization [47,48,49]. Some studies indicate that CYP1B1 expression modulates oxidative stress, activates nuclear factor-κB (NF-κB), and upregulates thrombospondin-2 (TSP2) in retinal vascular cells, influencing angiogenesis [48]. However, its role in retinal astrocytes, another major component of retinal vasculature, remains poorly understood.

Lastly, CYP1B1 is implicated in metabolic pathways relevant to glaucoma. It participates in the metabolism of vitamin A, crucial for embryonic eye development, and melatonin, a hormone with diverse ocular effects [42]. Specifically, CYP1B1 catalyzes retinol to retinal, the major forms of vitamin A, and further transforms the catalyzed retinal into retinoic acid [50]. Furthermore, CYP1B1 contributes to melatonin signaling, which has significant metabolic functions [51]. Melatonin synthesis in the eye, acting as a CYP1B1 inhibitor, and its modulation of retinal functions, such as dopamine synthesis, photoreceptor activity, and IOP regulation, suggest a potential link between CYP1B1-mediated retinoid metabolism, melatonin signaling, and glaucoma [52,53]. Moreover, CYP1B1 generates 12-(R)-HETE from arachidonic acid, serving as an endogenous corneal arachidonate metabolite [54,55]. Research by Masferrer et al. has shown that 12-(R)-HETE can effectively lower IOP in rabbits [55]. Additionally, lipidomic analyses have identified differences in endogenous metabolism within the eye between glaucoma patients and controls, indicating the potential involvement of metabolic pathways in the development of glaucoma [56]. These findings collectively suggest a potential link between CYP1B1-mediated metabolic pathways and the pathogenesis of glaucoma.

In summary, CYP1B1 plays multifaceted roles in PCG pathogenesis, influencing TM development, angiogenesis, and metabolic pathways crucial for ocular homeostasis. Further research is warranted to elucidate the precise mechanisms underlying its involvement in glaucoma and to explore its potential as a therapeutic target.

#### 2.1.3. CYP1B1 Animal Models

The expression pattern of Cyp1b1 in mice exhibits tissue-specific characteristics throughout development [57]. During early embryonic stages, Cyp1b1 mRNA is detectable in various regions, including the eye, hindbrain, brachial arches, forelimb bud, ligaments supporting the primordial liver, and kidney [58]. Subsequently, its expression becomes confined to the eye region and forelimb bud, with discernible alterations being observed in the neural retina as development progresses. Immunohistochemical studies in adult mice reveal the presence of Cyp1b1 in the eye, particularly in the ciliary and corneal epithelia, retinal cells, and ganglion cells [46]. Notably, developmental changes in Cyp1b1 protein levels are also observed in the lens epithelium. Studies on *Cyp1b1*^−/−^ mice, homozygous *Cyp1b1*-knockout (KO) mice, underscore the crucial role of constitutive Cyp1b1 expression in TM cells for their development and function [45]. Overall, the dynamic expression of cyp1b1 during both prenatal and postnatal development suggests its importance in guiding proper tissue development and function, particularly in the eye.

In 1999, a *Cyp1b1*-KO mouse line on the 129/Sv background was generated to elucidate the role of CYP1B1 in the metabolic activation of 7,12-dimethylbenz[α]anthracene [59]. Similarly, the role of CYP1B1 in PCG was investigated using Cyp1b1-null mice on a mixed 129 × 1/SvJ × C57BL/6J background, revealing defects reminiscent of those observed in PCG patients [60]. Notably, the severity of these defects was heightened in albino mice deficient in tyrosinase, an enzyme crucial for the production of melanin and l-dihydroxyphenylalanine (l-Dopa) [60]. Additionally, the symptoms were alleviated by administering l-Dopa, potentially through reducing oxidative stress. More recently, *Cyp1b1*-KO mice on a pure C57BL/6J background were used to explore the effects of Cyp1b1 on hypertension, revealing decreased TM collagen, elevated TM endothelial cell counts, and elevated collagen lesion scores compared to controls; these scores were observed to increase progressively with age [61]. 

Moreover, studies by Amirmokhtari et al. demonstrate that naive *Cyp1b1*-KO mice develop an anatomically intact retinal projection without overt signs of glaucomatous pathology [62]. However, following pressure elevation, accelerated degradation of axonal transport from the retina to the superior colliculus and altered morphology of the nodes of Ranvier and adjacent paranodes in the optic nerves were observed [62]. Their results suggest that the absence of Cyp1b1 expression alone may not be sufficient to induce glaucomatous pathology in mice. However, it may render retinal axons more susceptible to IOP elevation. Using a Cyp1b1-null mouse model, researchers have discovered that the tyrosinase gene (Tyr) acts as a modifier of the drainage structure phenotype, and the severe dysgenesis in eyes lacking both Cyp1b1 and Tyr can be alleviated via dihydroxyphenylalanine [60]. Additionally, in zebrafish, CYP1B1 indirectly influences neural crest migration by contributing to the closure of the ocular fissure [63]. Thus, selective upregulation of CYP1B1 expression in the eye holds promise for novel therapeutic strategies in PCG treatment. 

### 2.2. LTBP2

#### 2.2.1. *LTBP2* Mutations in PCG

**Figure 2:**
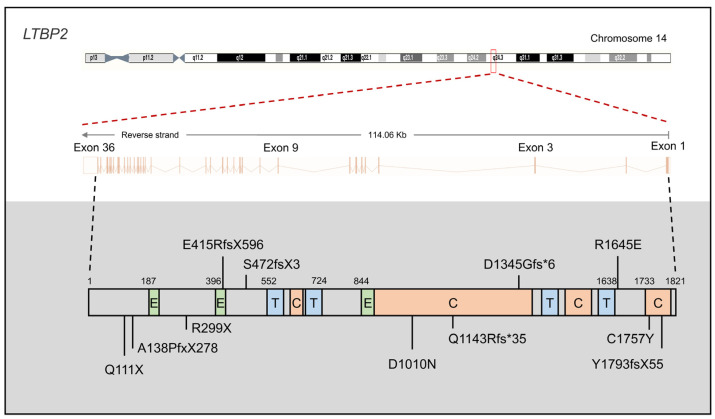
Gene structure of *LTBP2* and variants associated with primary congenital glaucoma (PCG). The *LTBP2* gene comprises 36 exons and codes a protein of 1821 amino acids, characterized by EGF-like (E), TB (T), and calcium-binding EGF-like (C) domains.

*LTBP2*, located on chromosome 14q24 within the *GLC3D* locus, is composed of 36 exons and encodes a matrix protein containing 1821 amino acids (aa) (Figure 2) [16]. In 2009, Ail et al. identified four homozygous null mutations in *LTBP2,* including p.Ala138fs*278 (p.A138PfsX278), p.Arg299*(p.R299X), p.Glu415fs*596 (p.E415RfsX596), and p.Gln111*(p.Q111X), as causative mutations of PCG in consanguineous families from Pakistan and individuals of Gypsy ethnicity [23]. Among these mutations, the truncating *LTBP2* p.Arg299* (p.R299X) mutation is the most prevalent Gypsy PCG mutation, accounting for approximately one-third of disease alleles (33.8%, from 34 families) [64]. In the same year, Narooie-Nejad et al. performed whole-genome autozygosity mapping in Iranian PCG families and identified two loss-of-function (LoF) mutations in *LTBP2* (p.S472fsX3 and p.Y1793fsX55) [24]. Subsequently, in 2016, Micheal et al. reported two novel mutations (p.R1645E and p.D1345Gfs*6) from consanguineous Pakistani PCG families using whole-exome sequencing [65]. Three novel *LTBP2* mutations (p.D1010N, p.Q1143Rfs*35, and p.C1757Y) were recently discovered in three consanguineous Pakistani PCG families [66].

#### 2.2.2. LTBP2 Protein Function in PCG

The LTBP2 protein plays a crucial role in tissue repair and cell adhesion, with ocular studies revealing its expression in both TM and ciliary processes [15]. Structurally, LTBP2 belongs to a superfamily of fibrillin and LTBP proteins, characterized by tandem arrays of epidermal growth factor-like motifs and interspersed TGFβ-binding protein-like motifs [67]. Unlike other isoforms, LTBP2 does not bind to latent TGFβ, suggesting functions independent of latent TGFβ storage and activation [68]. However, Hirani et al. reported an interaction between the C-terminal region of LTBP-2 and the N-terminal region of fibrillin-1, suggesting a role in regulating TGFβ activation by releasing LTBP-1 from microfibrils [69]. Considering the association of TGFβ with IOP [70], these results suggest the contribution of pathogenic *LTBP2* mutations to PCG through TGFβ-related mechanisms. 

Localization studies by Ail et al. confirmed the expression of LTBP2 in the anterior segment of the eye, particularly the ciliary process, indicating its importance in the normal development of the anterior chamber by maintaining ciliary muscle tone [23]. Moreover, these findings suggest that LTBP2 deficiencies may disrupt the elasticity of ciliary body structures, affecting the support provided to adjacent tissues such as Schlemm’s canal or the scleral spur [71]. Alternatively, alterations in the elasticity of the scleral spur could adversely affect the architecture of the TM, considering the anatomical connections between components of the ciliary body and the scleral spur [72]. Additionally, LTBP2’s involvement in tissue repair and cell adhesion suggests potential roles in maintaining microfibril and elastin fiber functions [24,73], as evidenced by its interactions with fibrillin-1 and fibulin 5 [74,75]. Despite these insights, the precise function of LTBP2 in PCG is yet to be fully elucidated. 

#### 2.2.3. LTBP2 Mouse Models

Investigating the in vivo functions of LTBP2 has posed a challenge, as the initial report of Ltbp2-null mice indicated a halt in development beyond embryonic day 6.5, indicating an essential role for LTBP2 in early embryogenesis [76]. Interestingly, PCG patients with homozygous nonsense LTBP2 mutations do not exhibit lethal birth defects or early developmental disorders, which presents a discrepancy between human and mouse models. However, this contradiction was recently addressed with the introduction of new Ltbp2-null mice, wherein exon 1 was deleted in the presence of Cre recombinase [75]. Remarkably, these mice were viable and fertile without any noticeable abnormalities. Detailed examinations revealed fragmented microfibrils in their ciliary zonules and dislocated lenses. Additionally, it was demonstrated that LTBP2 binds to fibrillin-1, and the addition of recombinant LTBP2 promotes the assembly of microfibril bundles in cultured cells and organ-cultured eyeballs. These findings underscore the significance of LTBP2 in eye development. However, further investigations are warranted to elucidate its role in PCG.

### 2.3. TEK and ANGPT1

**Figure 3:**
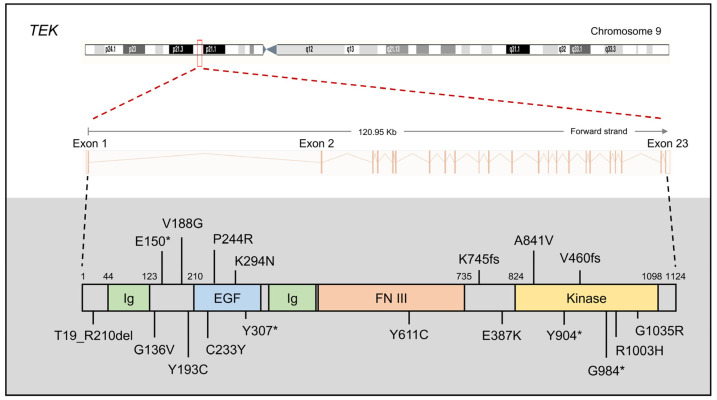
Gene structure of *TEK* and identified primary congenital glaucoma (PCG) variants. The *TEK* gene consists of 23 exons and codes a protein of 1124 amino acids, featuring Ig-like (Ig), EGF-like (EGF), fibronectin type III (FN III), and protein kinase (Kinase) domains. Variants reported in PCG patients are depicted, excluding those located in intronic regions: c.1624+5G>A, c.760+2T>C, and c.3300+2delT. Asterisk (*): premature stop codon.

**Figure 4:**
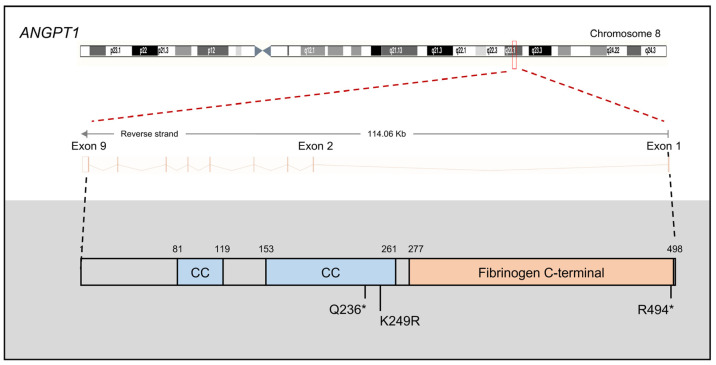
Schematic representation of the *ANGPT1* gene structure, protein domains, and three reported primary congenital glaucoma (PCG) mutations. The *ANGPT1* gene comprises nine exons and codes for a protein of 498 amino acids, characterized by two coiled-coil (CC) domains and one fibrinogen C-terminal domain. Asterisk (*): premature stop codon.

*TEK*, located on chromosome 14q24 within the *GLC3E* locus [21], is an integral component of the angiopoietin–TIE (tunica interna endothelial cell kinase) signaling pathway, which includes three ligands (ANGPT1, ANGPT2, and ANGPT4) and two receptors (TIE1 and TEK, also known as TIE2) [77]. Known as an endothelial growth factor pathway, the ANGPT–TIE system extends its involvement to processes such as inflammation, metastasis, and lymphangiogenesis [78]. Notably, heterozygous LoF variants in TEK (Figure 3) or its primary ligand ANGPT1 (Figure 4) have been associated with PCG [14,26]. 

#### 2.3.1. *TEK* and *ANGPT1* Mutations in PCG

The *TEK* gene comprises 23 coding exons encoding a principal product of 1124 aa. In 2016, Souma et al. first identified 10 heterozygous novel/rare *TEK* variants (p.T19_R210del, p.E150*, p.C233Y, p.K294N, p.Y307*, p.Y611C, p.K745fs, p.G984*, c.760+2T>C, and c.3300+2delT) as disease-causing mutations following dominant inheritance [14]. Seven of these mutations lead to abnormal LoF, including the absence of intact protein production, increased proteasomal degradation, impaired phosphorylation of critical tyrosine residue, changes in subcellular localization and diminished responsiveness to ligands, and protein aggregation. The remaining three mutations are also likely to be disease-associated. TEK could be implicated in the etiology of PCG, supported by their findings of the PCG-like phenotype in Angpt1/Angpt2 and Tek conditional KO mice [79]. In 2020, Young et al. identified eight *TEK* variants in families affected by PCG (p.G136V, p.Y904*, p.V188G, p.Y193C, p.P244R, p.A841V, p.G1035R, and c.1624 +5G>A), further supporting TEK’s role as a causative factor in PCG [80]. A subsequent study in China demonstrated that *TEK* mutations, particularly p.R1003H located in the protein kinase domain, may act as pathogenic mutation and contribute to PCG [81]. Taken together, these findings underscore the fact that defects in the ANGPT–TIE pathway caused by reduced TEK constitute a novel mechanism of the PCG disease. It is likely that investigating additional genes involved in forming and maintaining the ANGPT–TIE pathway could provide further insights.

Given the similarity in PCG-like phenotypes observed in *Angpt1*/*Angpt2* and *Tek* conditional KO mice, it has been hypothesized that other genes within the ANGPT–TIE system, such as *ANGPT1*, *ANGPT2*, and *ANGPT4*, might also play a role in PCG. In 2017, Thomson et al. identified heterozygosity for two novel nonsense variants (p.Q236*, p.R494*) and one rare missense variant (p.K249R) in *ANGPT1* in three PCG families without other known PCG-causing genes from a cohort of 284 PCG families [26]. The *ANGPT1* p.Q236* mutation introduces a premature stop codon within exon 4, resulting in a truncated protein lacking the C-terminal receptor-binding domain required for interactions with TEK [26]. The truncated ANGPT1 protein may have a dominant-negative effect on ANGPT1 signaling if it escapes the nonsense-mediated decay pathway. Additionally, Knight et al. identified an *ANGPT1* mutation in a cohort of 290 Australian PCG cases [4]. Furthermore, common variants in *ANGPT1* have been implicated as risk alleles for POAG in GWAS studies of 4986 cases and 58,426 controls from four racial/ethnic groups (non-Hispanic whites, Hispanics/Latinos, East Asians, and African Americans) [82]. 

#### 2.3.2. The Protein Function of TEK and ANGPT1

The TEK protein, identified as the ANGPT receptor discovered in 1992, form a small subfamily of growth factor receptor tyrosine kinases (RTKs) [77]. Its expression is primarily confined to the endothelium, including the endothelia of SC and collector channels, crucial components of the aqueous humor (AH) outflow pathway [83]. ANGPT1 is a paracrine ligand expressed by mesenchymal cells and acts as a potent TEK agonist, supporting endothelial cell survival, vessel stability, and endothelial barrier function [84]. The activation of TEK by ANGPT1 triggers downstream signaling through the protein kinase AKT (also known as protein kinase B), leading to the inhibition of transcription factor forkhead box protein O1 (FOXO1) and the suppression of FOXO1′s target genes, including *ANGPT2* [77]. 

Elevated IOP, a well-recognized key risk factor in PCG, is attributed to defects in the AH outflow pathway rather than increased AH production [14]. AH, produced by the ciliary body, drains mainly through SC and the uveoscleral pathways, and any disruption in fluid homeostasis can lead to elevated IOP [85,86]. The significant expression of the TEK receptor in the SC endothelium and the critical role of ANGPT–TIE signaling in SC development leads to significant outcomes when Tek or both major ANGPT ligands are deleted in mice after embryonic day 16.5. These outcomes include the developmental loss of SC, a notable increase in IOP, and a rapid and complete loss of retinal ganglion cells, all of which ultimately contribute to the development of glaucoma [79]. Thus, pathogenetic mutations in *TEK* and *ANGPT1* may contribute to elevated IOP and the development of PCG. 

#### 2.3.3. TEK and ANGPT1 Mouse Models

Since 2002, several mouse models of ocular disease related to the ANGPT–TIE system have been generated, including KO models for Angpt1 or Angpt2, conditional deletion of Angpt1, Angpt2, or Tek, as well as transgenic mouse models overexpressing retinal/ubiquitous ANGPT1 or ANGPT2 [77]. These models have provided insights into the role of the ANGPT–TIE system in retinal and choroidal neovascularization, particularly relevant to diseases affecting the outer retina and Bruch’s membrane, such as neovascular age-related macular degeneration (nAMD) [87]. 

Conditional deletion of Angpt1, or both Angpt1 and Angpt2, resulted in mice rapidly developing a severe glaucoma phenotype, characterized by the complete absence of SC, buphthalmos (enlargement of the eyeball), markedly elevated IOP, and severe damage to the neural retina [79]. The phenotype observed in mice lacking both Angpt1 and Angpt2 was more severe than that in mice lacking Angpt1 alone, with higher IOP levels of 35.58 ± 2.01 mmHg compared to 23.53 ± 1.50 mmHg at 8 weeks of age, suggesting compensatory mechanisms between ANGPT ligands within SC [26]. This severe phenotype was also replicated in mice lacking the Angpt receptor TEK. Conditional KO of Tek from embryonic day 17.5 resulted in the complete absence of SC and significantly elevated IOP, while heterozygous deletion of Tek led to a severely hypomorphic SC and increased IOP [14]. This dose–effect relationship clearly demonstrates the essential role of signaling through the classical ANGPT–TIE axis in SC formation [14,26]. 

### 2.4. FOXC1

The *FOXC1* gene, located on chromosome 6p25 and containing a single exon, encodes the FOXC1 protein, a member of the forkhead transcription factor family, which shares a highly conserved forkhead DNA-binding domain and plays essential roles in regulating various biological processes both during development and throughout adult life [88]. FOXC1 is essential for the development of multiple organs, including the eyes, skull, teeth, cardiovascular system, kidneys, and hematopoietic stem cells [88]. 

Mutations in *FOXC1* have been associated with anterior segment dysgenesis (ASD), particularly in Axenfeld–Rieger syndrome (ARS), globally [25]. ARS describes patients with Axenfeld–Rieger anomalies and additional systemic features, such as maxillary hypoplasia, teeth abnormalities, redundant umbilical skin, and congenital heart defects [89]. Diagnosis can be challenging in children, especially without a thorough ophthalmic examination under anesthesia [89]. Approximately 50% of individuals with ARS develop glaucoma, highlighting the significance of *FOXC1* in glaucoma pathogenesis [90]. Previous investigations have identified *FOXC1* mutations in PCG patients, suggesting the potential role of FOXC1 in PCG pathogenesis, particularly in cases where patients carry both *FOXC1* and *CYP1B1* alleles [25]. Recent findings have further supported this notion with the identification of rare pathogenic *FOXC1* variants in PCG cases without known CYP1B1 mutations, indicating that *FOXC1* sequencing could aid in PCG diagnosis, particularly when ARS is suspected [90]. In addition, some studies have implicated *FOXC1* in early-onset glaucoma associated with ASD and other ocular or non-ocular diseases [25].

One study using homozygous and heterozygous *Foxc1*-KO mice has demonstrated malformations in the anterior segment, leading to abnormalities in ocular drainage structures [91]. Additionally, a zebrafish model has shown that aberrant regulation of retinal ganglion cell (RGC) numbers may contribute to developing PCG and early-onset glaucoma due to *FOXC1* mutations [92]. 

## 3. Juvenile Open-Angle Glaucoma (JOAG)

Open-angle glaucoma, characterized by normal anatomic structures in the eye, typically develops in adults over 50, referred to as POAG. However, there are rare instances where this condition occurs in children or young adults before the age of 35, known as JOAG [3]. JOAG is inherited as an autosomal dominant trait and is characterized by extremely elevated IOP, often exceeding 50 mmHg, necessitating surgical treatment [16]. Several genetic loci have been associated with JOAG, including *GLC1A* (1q24.3–q25.2) [93], *GLC1J* (9q22) [94], *GLC1K* (20p12) [94], *GLC1M* (5q22.1–q32) [95], and *GLC1N* (15q22–q24) [96], with the *MYOC* gene identified within the *GLC1A* locus [97].

### 3.1. MYOC

#### 3.1.1. *MYOC* Mutations in JOAG

**Figure 5:**
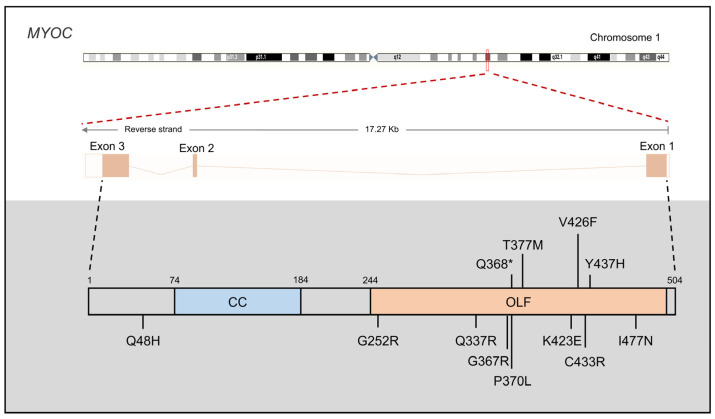
Gene structure of *MYOC* and reported 12 primary congenital glaucoma (PCG) variants by multiple independent research groups. The *MYOC* gene consists of 3 exons and encodes a protein of 504 amino acids, including one coiled-coil (CC) and one olfactomedin-like (OLF) domain. Asterisk (*): premature stop codon.

The *MYOC* gene, also known as the TM-inducible glucocorticoid response (TIGR) gene, is located on chromosome 1q24 and contains three exons. In 1997, Stone et al. initially linked *MYOC* mutations to JOAG [97]. While *MYOC* mutations can lead to both JOAG and adult-onset POAG, the majority of these mutations result in JOAG, often manifesting as a familial disease [2]. Currently, over 250 *MYOC* mutations have been reported, of which 37.7% are considered pathogenic [98]. Notably, approximately 97% of disease-causing *MYOC* mutations localize to exon 3, which encodes the olfactomedin homology domain (myocilin allele-specific phenotype database: myocilin.com, accessed on 24 January 2024). Although most pathogenic *MYOC* mutations are missense alleles (84%), the most common *MYOC* mutation identified in JOAG is a nonsense mutation known as p.Gln368stop, causing protein aggregation [98]. In addition to being a prevalent JOAG-causing mutation, *MYOC* p.Pro370Leu is associated with severe pathology [99], and *MYOC* p.Tyr437His is correlated with the form of glaucoma characterized by an earlier age of onset and significantly elevated IOP levels [100]. Moreover, nine additional *MYOC* mutations have been identified by multiple independent research groups (Figure 5). Furthermore, certain *MYOC* mutations, including p.N57D [101], p.C245Y [102], p.P274R [103], p.I345M [104], p.T377R [105], p.D384H [106], p.E385K [107], p.Y453Mfs*11 [104], and p.I499S [101], have been exclusively reported by single research teams.

#### 3.1.2. MYOC Protein Function in PCG

MYOC, a secreted glycoprotein, is expressed in various eye structures, including the retina, ciliary body, and TM, with the highest mRNA level detected in TM tissue [108,109,110,111]. As well as the expression of MYOC is also demonstrated in skeletal muscles, heart, brain, and testes [112]. Despite its widespread expression, MYOC does not appear essential for ocular health, as evidenced by both KO animal models and individuals with homozygous, likely null mutations who do not exhibit glaucoma phenotypes [113,114]. Notably, the expression of MYOC is influenced by several molecules, including steroids, TGF-ꞵ1, and the protein optineurin, particularly within cultured TM cells [115]. 

Molecular and biochemical studies have elucidated that mutated MYOC tends to accumulate in the endoplasmic reticulum (ER) rather than proper release, leading to activation of the unfolded protein response (UPR) and subsequent ER stress [112,116]. Under normal circumstances, the UPR triggers reduced translation and increased chaperone levels to aid in proper protein folding and secretion [117]. However, in cases of overwhelming misfolding, cell apoptosis may ensue [117]. TM cells, particularly sensitive to prolonged ER stress, may eventually perish, thereby contributing to elevated IOP and the development of glaucoma in individuals with *MYOC* mutations [112].

#### 3.1.3. MYOC Mouse Models

Various research groups have utilized mouse genetics to investigate the role of MYOC in both normal and pathological retinal functions. In both 2001 and 2004, Kim et al. and Gould et al., respectively, demonstrated that MYOC mutations are gain-of-function mutations, as neither KO nor transgenic Myoc mouse model did not lead to a glaucoma phenotype or elevated IOP [114,118]. Consequently, subsequent investigations focused on examining the disease-specific MYOC p.Y437H mutation in mice, responsible for a severe form of JOAG [100]. Initially, expression of human MYOC p.Y437H and the corresponding mouse Myoc mutation in mouse lens did not lead to elevated IOP or RGC loss [3]. Until 2007, Shepard et al. crucially observed elevated IOP upon adenovirus-mediated expression of human MYOC p.Y437H in the iridocorneal angle [119]. Notably, human MYOC contains a peroxisome targeting signal absent in mouse Myoc, which is critical for mutant MYOC induced toxicity in TM cells; thus, the corresponding mouse Myoc did not develop the glaucoma phenotype. Subsequent studies from independent groups confirmed IOP elevation using various strategies to express human MYOC p.Y437H in mouse eyes, suggesting alternative pathogenic mechanisms for other MYOC mutations [120].

## 4. Discussion

Currently, six genes are known to be implicated in childhood primary glaucoma, with mutation carriers often exhibiting variable phenotypes. These genes display diverse inheritance patterns: *CYP1B1* and *LTBP2* follow an autosomal recessive pattern, while *TEK* and *ANGPT1* adhere to autosomal dominant inheritance. *FOXC1* and *MYOC* mutations are detected in both autosomal dominant cases and sporadic PCG instances. The prevalence of *CYP1B1* mutations in PCG varies significantly across populations, with higher frequencies seen in regions characterized by high consanguinity [121]. Similarly, most *LTBP2* mutations leading to PCG are concentrated in areas with pronounced consanguinity, notably Pakistan and Iran. Numerous genetic and molecular studies have provided insights into the biological processes underlying ophthalmic disorders. This review focused on these gene-associated mutations in childhood primary glaucoma, their protein function, and relevant mouse models.

Childhood glaucoma forms typically manifest with elevated IOP. Nevertheless, familial instances of normal-tension glaucoma also exist [122]. *OPTN*, *TBK1*, and *METTL23* are responsible for early onset normal-tension glaucoma with autosomal dominant inheritance, characterized by significant optic atrophy despite normal IOP [70,123]. *OPTN* and *TBK1* are pivotal in critical cellular processes, notably autophagy and NF-kB signaling, with the encoded proteins known to interact [123]. In addition, METTL23 catalyzes H3R17 dimethylation in the retina, which is critical for RGC homeostasis by negatively regulating NF-κB–mediated TNF-α and IL-1β feedback [70]. These findings suggest that genes encoding NTG-related proteins or those involved in autophagy and NF-kB signaling may also contribute to childhood glaucoma.

Furthermore, PCG is classified under ASD, characterized by congenital improper development of anterior ocular tissues [120]. ASD, in any form, elevates the risk of childhood glaucoma by 50% [124]. Subtle anterior segment abnormalities in early childhood significantly contribute to later-in-life IOP elevation, independent of clinical severity [125]. The multifactorial etiology involves genetic variants, which remain incompletely understood at the cellular and molecular levels despite advances in sequencing technology. Continued research is crucial for identifying gene functions in normal development and understanding how mutations contribute to ASD, enabling the development of diagnostic and screening tests to identify at risk individuals before irreversible optic nerve damage occurs.

Moreover, primary childhood glaucoma patients may have pathogenetic mutations in one or more causing genes, with potential regulatory or interactional relationships among them. *CYP1B1* may act as a modifier gene for MYOC expression, and a digenic mode of inheritance involving *CYP1B1* and *MYOC*, as well as *CYP1B1* and *TEK*, has been considered [126]. Currently, genetic testing yields a 40% chance of identifying a genetic cause [127]. Further exploration into the intricate genetic mechanisms underlying PCG is imperative for enhancing diagnostic accuracy and developing targeted therapies to manage the condition and improve patient outcomes effectively.

## Data Availability

Not applicable.
